# Adalimumab to treat noninfectious pediatric chronic anterior uveitis: a case series

**DOI:** 10.1007/s10792-024-03289-1

**Published:** 2024-09-10

**Authors:** Raul E. Ruiz-Lozano, Hazem M. Mousa, Matias Soifer, Nadim S. Azar, Manuel E. Quiroga-Garza, Daliya Dzhaber, Sofia Murillo, Ailin Song, C. Egla Rabinovich, Victor L. Perez

**Affiliations:** 1 Department of Ophthalmology, Foster Center for Ocular Immunology at Duke Eye Center, Duke University School of Medicine, Durham, NC USA; 2 Bascom Palmer Eye Institute, University of Miami, Miami, FL USA; 3 Department of Pediatrics, University of California, San Francisco, San Francisco, CA 94143 USA; 4 Department of Pediatrics, Duke University Health System, Durham, NC 27705 USA; 5 McKnight Vision Research Center, Bascom Palmer Eye Institute, University of Miami Miller School of Medicine, 900 NW 17th St, Miami, FL 33136 USA

**Keywords:** Adalimumab, Anti-adalimumab antibodies, Chronic anterior uveitis, Juvenile idiopathic arthritis, Pediatric uveitis, Uveitis recurrence

## Abstract

**Purpose:**

Evaluate the response to adalimumab (ADA) in pediatric chronic anterior uveitis (pCAU).

**Methods:**

Retrospective chart review of pCAU patients treated with ADA. Outcomes evaluated included the proportion of patients achieving zero ocular inflammation and discontinuation of topical corticosteroids, visual outcomes, and incidence of uveitis recurrences after ≥ 12 months of prescribing ADA. Incidence and risk factors for developing anti-adalimumab antibodies (AAAs) were also evaluated.

**Results:**

Of 27 children aged 11 years, 16 (59%) were Caucasian and 6 (22%) African Americans. Thirteen (48%) patients had idiopathic pCAU, 12 (44%) had juvenile idiopathic arthritis (JIA) related pCAU, and 2 (7%) had tubulointerstitial nephritis and uveitis syndrome. At baseline, African American children had worse visual acuity (*p* = 0.026). At 1 year, 21 (78%) children achieved zero ocular inflammation (remission). Risk factors associated with non-remission were being African American (20% vs. 94%, *p* = 0.003) and experiencing ≥ 1 episode of uveitis recurrence (100% vs. 0%, *p* < 0.001). Six episodes of uveitis recurrence were documented in five children, four of whom were African American. Topical corticosteroids were discontinued in 83% of children, and visual acuity remained stable for 1 year. Twelve children were tested for AAAs due to arthritis or uveitis flare-ups, with five (42%) being positive. No significant factors were associated with the development of AAAs.

**Conclusions:**

We found that ADA is effective in controlling inflammation, reducing the need for topical corticosteroids, and maintaining visual acuity in pCAU. There appears to be racial differences in African American children who had worse baseline disease and poorer outcomes. Studies are necessary to understand better and address these disparities.

## Introduction

Pediatric chronic anterior uveitis (pCAU) is an ocular inflammatory condition that remains a diagnostic and therapeutic challenge [1]. Uveitis is a common manifestation of systemic autoimmune diseases in children. For example, in juvenile idiopathic arthritis (JIA), uveitis is the most frequent extra-articular manifestation developing in 12–38% of cases [2]. In JIA, inflammation is usually chronic and asymptomatic, although it may present as acute anterior uveitis, characterized by sudden episodes of unilateral pain, erythema, and photophobia [3]. Without appropriate diagnosis and management, inflammation can progress into irreversible vision loss. Historically, 25% of children with JIA-uveitis were legally blind in at least one eye at the time of presentation [4, 5]. The management of pCAU is challenging due to difficulties screening this population and a limited therapeutic arsenal. Treatment alternatives, which must target the local and systemic inflammatory process, include corticosteroids, disease modifying antirheumatic drugs (DMARDs), and biologic agents.

Among the biologic agents, studies report that adalimumab (ADA) is safe and effective in pediatric uveitis [3]. ADA is a humanized monoclonal antibody that inhibits tumor necrosis factor (TNF)-alpha, an inflammatory cytokine involved in the pathogenesis of uveitis [3]. Clinical trials have shown ADA to have a better safety and efficacy profile than other anti-TNF inhibitors (i.e., infliximab and etanercept) for managing pediatric uveitis. Thus, it is the only anti-TNF-alpha inhibitor approved by the Food and Drug Administration (FDA) for use in uveitis [6, 7].

Unfortunately, as with all biologic drugs, the use of ADA can have varying success rates and is susceptible to the development of neutralizing anti-drug antibodies which can be associated with a reduced circulating drug concentration and lower overall efficacy [6]. Anti-adalimumab antibodies (AAAs) develop in ~ 14% of cases; however, the latter may vary depending on the underlying cause of the uveitis [6, 8, 9]. For example, while two randomized controlled trials evaluating the efficacy of ADA in adult patients with non-infectious uveitis of varying etiologies report a 2.7% and 5% incidence of AAAs, respectively, another prospective study with patients of similar characteristics reported a 32% incidence of AAAs [10–12]. In patients with JIA-associated uveitis, the incidence of AAAs ranges from 16 to 45% [13–15]. Most JIA patients develop AAAs after 3 months of ADA therapy, 60% of AAA-positive patients require a drug switch due to therapeutic failure at 1 year, and permanent AAA is associated with loss of clinical response and vision loss [13]. Unfortunately, there is a paucity of data regarding the prevalence of AAAs among children with non-JIA-uveitis.

This study assessed the demographic characteristics, clinical outcomes, and risk factors for AAAs development in pCAU patients managed with ADA from Duke Eye Center.

## Methods

### Study design and population

We conducted a single center, retrospective chart review of children with chronic, non-infectious uveitis seen at the Pediatric Uveitis Multidisciplinary Clinic in the Foster Center for Ocular Immunology at the Duke Eye Center between July 2018 and July 2022. Patients were eligible if they were ≤ 18 years old when the uveitis was diagnosed and received ADA as part of their uveitis treatment regimen for at least 6 months or more. Patients with uveitis and a systemic autoimmune disease who have been prescribed ADA for non-uveitis reasons were excluded. The Institutional Review Board approved this study at Duke University Hospital.

### Patient characteristics

An extensive clinical history, physical examination, and serologic workup to exclude any potential underlying infection or systemic autoimmune disease responsible for uveitis were performed in all patients. Before administering ADA, all children were screened for chronic infectious diseases, including latent tuberculosis. Periodic (12 weeks) laboratory tests, including complete blood count, complete metabolic panel, and urinalysis, were performed in all cases to monitor for potential adverse effects. Patients received 10–40 mg of subcutaneous ADA every two weeks, increasing according to treatment response (maximum dose: 40 mg). Doses were not modified when ADA was initiated for children under treatment with systemic steroids and other immunosuppressants. However, systemic and topical steroids were tapered according to clinical response. At each visit, all children were evaluated simultaneously by the pediatric rheumatologist (C.E.R) and ocular immunologist (V.L.P) for treatment response and development of any systemic or ocular complication. Both specialists determined the frequency of visits according to disease severity and response to therapy.

### Data collection

The following baseline data were retrieved from medical records: demographics (age, gender, ethnicity), weight, uveitis characteristics (type, duration, laterality), ocular comorbidities (glaucoma, band keratopathy, cataract), presence of other systemic diseases, human leukocyte antigen (HLA)-B27 status and antinuclear antibody (ANAs) levels when available, and previous use of systemic steroids, immunosuppressants, or biologics. The number of documented uveitis flare-ups in the 2 years before ADA was also recorded. The corrected distance visual acuity (CDVA) was measured with the Snellen chart and converted into a logarithm of the minimum angle of resolution (LogMAR) units. A BCVA below 20/400 was converted as follows: counting fingers = 2.0; hand motion perception = 2.3; light perception = 2.6; and no light perception = 2.9 [16]. A complete slit lamp and binocular indirect ophthalmoscopic examination with pupillary dilation assessing for corneal transparency, anterior chamber (cell count) and posterior (vitreous haze) chamber inflammation, intraocular pressure measurement, lens status, cup-to-disc ratio, and cystoid macular edema (CME) were performed at every visit. ADA immunogenicity was evaluated for patients with uveitis and/or arthritis recurrence through serum AAAs levels, measured by bridging enzyme-linked immunosorbent assay (ELISA).

### Study definitions and outcome measures

The outcomes of this study included the proportion of children who (1) achieved inflammatory control, defined as zero ocular inflammation, (2) experienced a uveitis recurrence, (3) achieved remission, and (4) the change in visual acuity. Separately, we analyzed children who developed AAAs and the potential patterns of association. Inflammatory control (inactive disease) was defined using the Standardization of Uveitis Nomenclature (SUN) as the presence of < 1 (grade 0) anterior chamber cells in a 1 mm by 1 mm slit beam field size after ADA initiation in both eyes [17]. Remission was defined as sustained inflammatory control without needing topical steroids for at least 3 months. Finally, uveitis recurrence was defined as new-onset anterior chamber inflammation after achieving complete inflammation resolution, thus requiring adding or increasing topical corticosteroids with or without an increase in systemic therapy [17].

### Statistical analysis

Statistical analyses were performed using IBM SPSS V.25 (IBM). The Shapiro-Wilks test was used to evaluate normality. Normally distributed data were described with means and standard deviations (SDs), whereas skewed data with medians and interquartile ranges (IQRs). The chi-square or Fisher exact test was used to compare categorical variables, and the Wilcoxon rank-sum paired t-test was used to compare means. The analysis of repeated measures was performed through analysis of variance (ANOVA) and paired t-test. The statistical significance was set at a *p* value < 0.05.

## Results

### Patient characteristics

Of 49 patients with pediatric uveitis that were seen at the Pediatric Uveitis Multidisciplinary Clinic in the Foster Center for Ocular Immunology during the study period, 27 (55.1%) completed ≥ 6 months of follow-up after ADA was prescribed and, thus, were included. The baseline demographic and clinical characteristics are listed in Table 1. Prior ocular complications (one or more) were present in 22 (81.5%) children, including synechiae (11 children), cataracts (9 children), band keratopathy (9 children), secondary glaucoma (4 children), CME (4 children), and epiretinal membrane (1 child). Fourteen (51.9%) children had systemic inflammatory disease, including JIA (12 children) and tubulointerstitial nephritis and uveitis syndrome (TINU, 2 children).

**Table 1:** Baseline characteristics of pediatric patients with uveitis at initiation of adalimumab therapy

Variable	No. patients (%) (n = 27)
*Gender*	
Female	20 (74.1)
Male	7 (25.9)
Median age (Q1, Q3), years	11 (15, 6)
*Ethnicity*	
Caucasian	16 (59.3)
African American	6 (22.2)
Asian	1 (3.7)
Other	4 (14.8)
*Type of uveitis*	
Anterior	25 (92.6)
Intermediate	2 (7.4)
*Previous uveitis flares*^*a*^	
< 3	21 (77.8)
≥ 3	6 (22.2)
*Systemic diagnoses*	
None/idiopathic	13 (48.1)
Juvenile idiopathic arthritis	12 (44.4)
Tubulointerstitial and uveitis syndrome	2 (7.4)
*JIA subcategory (n* = *12)*	
Oligoarticular	7 (58.3)
Psoriatic	1 (8.3)
Enthesitis related	2 (16.7)
Polyarticular	2 (16.7)
*Laboratory tests*	
Positive ANA results	16 (59.3)
Positive HLA-B27 results	5 (18.5)
Median duration of uveitis (IQR), months	19 (24)
*Baseline therapy*^*b*^	
Topical glucocorticoids	26 (96.3)
Oral glucocorticoids	5 (18.5)
Methotrexate	17 (63.0)
Leflunomide	1 (3.7)

IQR, interquartile range; JIA, juvenile idiopathic arthritis; ANA, antinuclear antibody; HLA, human leukocyte antigen

^a^Includes uveitis flares in either eye in the previous 2 years

^b^May include one or more

Table 2 presents the treatment details after ADA was prescribed and up to the first year of follow-up. At the initiation of ADA treatment, 17 (63.0%) children were taking MTX at a median weekly dose of 20 mg (range, 7.5–25 mg). Eleven children were taking MTX by subcutaneous injection and 6 by mouth. ADA and MTX were initiated concomitantly in 3 patients due to severe ocular inflammation (patients 11, 16, and 20). All but one child (96.3%) with no ocular inflammation were using topical corticosteroids, and 5 (18.5%) were using oral prednisone (10 to 20 mg daily) at ADA initiation (patients 2, 3, 5, 6, and 7). Patient 4 was under treatment with leflunomide (10 mg per day) due to previous gastrointestinal intolerance to MTX.

**Table 2:** Treatment details and relapse of 27 patients with pediatric chronic anterior uveitis managed with ADA after 1 year of follow-up

Pt No	Systemic disease	At ADA initiation					At inactive disease (inflammatory control)					Relapse Uveitis	AAAs (tested/ result)
		AC Cells (OD/OS)	Therapy				Inactive uveitis in OU (visit)	Therapy					
			Topic CS	PRD (mg/d)	MTX (mg/wk)	ADA (dose)		Topic CS	PRD (mg/d)	MTX (mg/wk)	ADA (dose)		
1	Idiopathic	0 +/1 +	+	–	25 SQ	40/q2w	6 months	+	–	25 SQ	40/q2w	–	N/A
2	JIA	1 +/1 +	+	10	20 SQ	20/q2w	3 months	+	–	25 SQ	20/q1w	+	N/A
3	Idiopathic	0.5 +/1 +	+	20	15 PO	40/q2w	3 months	+	+	12.5 PO	40/q2w	+	+/−
4	JIA	1 +/0.5 +	+	–	10 mg/d^a^	20/q2w	3 months	–	–	20 mg/d^a^	40/q2w	–	+/−
5	JIA	1 +/0.5 +	+	15	25 SQ	40/q2w	6 months	+	+	25 SQ	40/q2w	–	N/A
6	TINU	1 +/2 +	+	20	–	40/q2w	3 months	+	+	17.5 SQ	40/q1w	+	+/−
7	Idiopathic	2 +/2 +	+	10	20 SQ	20/q2w	6 months	+	–	25 SQ	40/q2w	–	+/−
8	JIA	0.5 +/2 +	+	–	5 PO	40/q2w	3 months	–	–	5 PO	40/q1w	–	+/−
9	JIA	1 +/0.5 +	+	–	7.5 PO	40/q2w	3 months	+	–	7.5 PO	40/q2w	–	N/A
10	Idiopathic	1 +/0.5 +	+	–	–	40/q2w	3 months	–	–	–	40/q2w	–	N/A
11	Idiopathic	0 +/2 +	+	–	25 SQ^b^	40/q2w	3 months	+	–	25 SQ	40/q1w	–	N/A
12	Idiopathic	2 +/0 +	+	–	20 SQ	20/q2w	3 months	–	–	10 SQ	20/q2w	–	N/A
13	JIA	0.5 +/2 +	+	–	25 SQ	20/q2w	3 months	–	–	12.5 SQ	40/q2w	–	N/A
14	TINU	1 +/0.5 +	+	–	–	40/q2w	3 months	–	–	10 SQ	40/q1w	–	N/A
15	Idiopathic	2 + ^c^/2 + ^c^	+	–	–	20/q2w	3 months	+	–	–	40/q2w	–	N/A
16	Idiopathic	0.5 +/1 +	+	–	5 SQ^b^	20/q2w	3 months	–	–	5 SQ	20/q2w	–	N/A
17	JIA	1 +/0.5 +	+	–	12.5 SQ	10/q2w	3 months	+	–	12.5 SQ	40/q2w	–	+/−
18	Idiopathic	0.5 +/1 +	+	–	15 PO	40/q2w	6 months	–	–	15 PO	40/q2w	–	N/A
19	Idiopathic	2 +/2 +	+	–	20 SQ	20/q2w	3 months	–	–	20 SQ	40/q2w	–	N/A
20	Idiopathic	0.5 +/1 + ^c^	+	–	7.5 PO^b^	40/q2w	6 months	–	–	7.5 PO	40/q1w	–	N/A
21	JIA	1 +/1 +	+	–	–	40/q2w	9 months	–	–	5 SQ	40/q1w	–	+/−
22	Idiopathic	1 +/1 +	+	–	25 SQ	40/q2w	6 months	+	–	25 SQ	40/q2w	+	N/A
23	JIA	1 +/1 +	+	–	20 SQ	40/q2w	6 months	–	–	7.5 SQ	40/q2w	–	+/+
24	JIA^d^	0.5 +/0.5 +	–	–	–	40/q2w	3 months	–	–	20 mg/d^a^	40/q1w	–	+/+
25	Idiopathic	0.5 +/2 +	+	–	15 PO	40/q2w	3 months	–	–	–	40/q2w	–	+/+
26	JIA^d^	0.5 +/0.5 +	+	–	17.5 PO	40/q2w	3 months	+	–	15 SQ	40/q2w	+	+/+
27	JIA	2 +/2 +	+	–	25 PO	40/q2w	3 months	+	–	25 SQ	40/q2w	–	+/+

ADA, adalimumab; Pt No, patient number; AC, anterior chamber; OD, right eye; OS, left eye; CS, corticosteroids; PRD, prednisone; MTX, methotrexate; OU, both eyes; AAAs, anti-adalimumab antibodies; SQ, subcutaneous; q2w; every two weeks; N/A, not applicable; JIA, juvenile idiopathic arthritis; q1w, every week; PO, by mouth; TINU, tubulointerstitial nephritis and uveitis syndrome

^a^Indicates oral leflunomide

^b^Indicates patients in whom MTX and ADA were initiated concomitantly

^c^Indicates eyes with vitreous cells. ^d^Indicates two patients who were initially managed with ADA for arthritis but developed ocular inflammation during follow-up

### Response to adalimumab (ADA) treatment

The median initial ADA dose was 40 mg (range, 10–40 mg). Injections were initially administered every two weeks, with dosing increased to weekly, if necessary, until ocular inflammation was under control. Dosing adjustments were determined by the treating ophthalmologist (V.L.P) and pediatric rheumatologist (C.E.R). Once achieving < 1 cells (grade 0), systemic and topical corticosteroids were tapered. Oral corticosteroids were discontinued in three out of five children at 6 months and all children at 1 year follow-up. MTX was stopped in three children, and the dose was reduced in five patients due to intolerance. There were no severe adverse effects or serious infusions reactions in all but one patient (patient 21) who developed an inguinal methicillin-resistant *Staphylococcus aureus* infection requiring drug discontinuation. However, ADA was reintroduced once the condition was resolved.

After 6 months of follow-up, 24 (88.9%) of 27 children had no anterior chamber inflammation, and 17 (63.0%) were off topical corticosteroids (Table 3). The CDVA remained stable, with no significant changes during the first year of follow-up (Table 4). One child underwent cataract extraction during treatment. At the last visit, five eyes from three patients had a visual loss of ≥ 2 Snellen lines compared to baseline due to worsening cataracts or band keratopathy.

**Table 3:** Percentage of children achieving zero ocular inflammation and discontinuing topical corticosteroids after 3, 6, 9, and 12 months of initiation of adalimumab

	Baseline	3 months	6 months	9 months	12 months
No. patients (%) with zero ocular inflammation	2 (7.4)	19 (70.4)	24 (88.9)	21 (87.5)	18 (78.3)
Comparison with baseline, *P* value	N/A	< 0.001	< 0.001	< 0.001	< 0.001
No. patients (%) off topical corticosteroids	1 (3.7%)	10 (37.0)	17 (63.0)	18 (75.0)	19 (82.6)
Comparison with baseline, *P *value	N/A	0.001	< 0.001	< 0.001	< 0.001
Total no. of patients with available data	27	27	27	24	23

N/A, not applicable

**Table 4:** Change in visual acuity during follow-up after initiation of adalimumab

	Baseline	3 months	6 months	9 months	12 months
Mean CDVA (LogMAR)	0.248	0.218	0.213	0.256	0.295
Total no. of patients with available data	27	24	24	23	23
Comparison with baseline, *P* value	N/A	0.286	0.720	0.345	0.808

CDVA, corrected distance visual acuity; LogMAR, logarithm of the minimum angle of resolution; N/A, not applicable

Six episodes of uveitis recurrence were documented in five (21.7%) of 23 children who completed 12 months of follow-up (patients 2, 3, 6, 22, and 26). In two patients, the uveitis recurred during a dose-reduction attempt of ADA, in another two, due to patients’ failure to comply with therapy, and in the remaining case, because of AAAs development (patient 26). At the discretion of the pediatric rheumatologist and ophthalmologist, uveitis recurrences were managed with one or more of the following interventions: change in frequency of topical corticosteroid use, a short course of systemic corticosteroids, and reintroduction or increased dosing of ADA with or without methotrexate or switching to another inhibitor of TNF-alpha (infliximab).

### Factors associated with remission

Baseline and 1 year data were available in 23 of 27 children. Of those, 18 (78.3%) were in remission at the last visit. African American (1/5 patients) children were less likely to achieve remission than non-African American (17/18 patients, *p* = 0.003). Recurrence of uveitis was associated with non-remission at 1 year (100% vs. 0%, *p* < 0.001). Other variables, including gender, age, type of uveitis, baseline ocular complications, systemic diagnoses, and duration of uveitis before ADA therapy, were not associated with achieving remission at 1 year.

### Anti-adalimumab antibody (AAA) testing

The 27 patients with ADA therapy completed a median follow-up time of 31 months (range, 22–47 months). At the last visit, 20 (74.1%) children were still using ADA, and 7 (25.9%) required drug discontinuation due to AAAs development (five cases) or loss of efficacy. During follow-up, 12 patients were tested for AAAs because of uveitis or arthritis flare-ups. Among the positive cases (5 children, 41.7%), two were switched to infliximab, two to certolizumab, and one to MTX (Table 5). There were no variables significantly associated with positive AAAs.

**Table 5:** Treatment details in 5 patients developing AAAs

Pt no	Time elapsed between ADA initiation and AAAs testing	Reason for AAAs testing	Therapy after positive AAAs
23	3 months	Increasing ocular inflammation	Switched adalimumab for infliximab and continued with SQ methotrexate (20 mg/week)
24	36 months	Arthritis flare-up	Switched adalimumab for infliximab and continued with leflunomide (20 mg/day)
25	20 months	Uveitis flare-up	Switched adalimumab for SQ methotrexate (25 mg/week)
26	9 months	Uveitis flare-up	Switched adalimumab for abatacept. Due to insurance problems, abatacept was switched for certolizumab and the dose of SQ methotrexate was increased (25 mg/week)
27	27 months	Uveitis flare-up	Discontinued adalimumab and switched PO methotrexate for SQ methotrexate (25 mg/week). 1 year later, the patient had another uveitis flare-up controlled with topical corticosteroids. 3 years later, the patient had a new arthritis flare-up managed with certolizumab

AAAs, anti-adalimumab antibodies; Pt No, patient number; ADA, adalimumab; SQ, subcutaneous

## Discussion

In the present series, ADA was safe and effective in managing 27 pCAU patients with a median follow-up of ≥ 2 years. Of those, 63% of children had active uveitis despite treatment with high-dose MTX, with more than half presenting with ocular complications. According to a meta-analysis, MTX is safe and effective in children with uveitis, achieving remission in 73% of patients at a median time of 3.5 months (range, 1–12) [18]. However, lack of efficacy and adverse effects, including gastrointestinal disturbance and elevated liver enzymes, can lead to therapy discontinuation and uveitis recurrence [18]. At this point, recommendations by the American College of Rheumatology/Arthritis Foundation Guidelines (ACR/AFG) suggest initiating therapy with a monoclonal antibody TNF-alpha inhibitor such as ADA [3]. In our study population, 78% of children had zero ocular inflammation, 83% were off topical corticosteroids, and visual acuity remained stable after 1 year of ADA therapy. Similar results were reported by a multi-center randomized study of children with JIA-related uveitis refractory to MTX, showing that adding ADA (at a dose of 20–40 mg every two weeks) was significantly associated with fewer treatment failures compared to placebo (27% vs. 60%, *p* < 0.0001) [19].

Most of our pCAU patients were Caucasian (59%) or African American (22%), in line with the census demographic data on the population of North Carolina. Of interest, race appeared to be a focal determinant of outcomes in our study. Specifically, children in the non-remission group were more likely to be African American compared to the remission group. In addition, most of the patients that had relapse of uveitis were also African American. These findings are consistent with previous reports of racial disparities in the incidence and outcomes of uveitis [20, 21]. A cross-sectional study evaluating more than 90 million discharges from the US National Inpatient Sample reported that odds of uveitis and ocular complications were significantly higher in African Americans compared to Caucasians [21]. In a chart review of 85 children with uveitis (52 [55%] with JIA-associated uveitis and 42 [45%] with other types of uveitis), Angeles-Han et al. reported that African Americans were older at uveitis onset (9.2 vs. 5.3 years, *p* = 0.03), had a worse visual acuity at baseline (median, 0.40 vs. 0.14, *p* = 0.014), and an increased prevalence of ocular complications such as band keratopathy (59% vs. 18%, *p* < 0.001) and cataracts (67% vs. 31%, *p* = 0.004) compared with Caucasians [20]. Risk factors for developing chronic uveitis in children with JIA are well known. The American Academy of Pediatrics [22] and the American College of Rheumatology [3] established guidelines for the screening of uveitis in children with JIA. Angeles-Han et al. [20] also found that the uveitis in African Americans was less likely to be secondary to JIA (*p* = 0.019). This finding was supported by another study reporting that African Americans had a lower risk of developing JIA-associated uveitis [23]. In our study, African Americans also had a significantly worse visual acuity at baseline and a non-significant trend towards an increased prevalence of cataracts and synechiae compared with non-African Americans. The fact that JIA-related uveitis is less prevalent in African Americans could potentially lead to a later uveitis diagnosis in this population through screening programs [20, 24]. However, the determinants of such differences between different racial groups remain not fully understood.

On the immunologic end, Nussenblatt and Mittal reported that the phenotypic frequency of the HLA-B8 antigen was significantly higher among healthy African-Americans than among Caucasians [25]. Of interest, the authors also found that African-Americans with uveitis and positive for the HLA-B8 antigen had worse visual outcomes than those from the HLA-B8 negative group [25]. Nonetheless, race is a complex construct, and it is important to consider the relevance of such genetic factors in a much larger context that involves social and system-based determinants. Studies aiming to understand and thus address better these racial differences are necessary to improve outcomes. African American children in this sample presented at an older median age than non-African American patients and had a significantly worse baseline CDVA, suggesting that disparities in access to healthcare and specialized therapies in this population could be in play behind the differences in outcomes. This is particularly true considering that African Americans have been reported to be the subjects of disparities in healthcare across a wide spectrum of medical conditions [26]. Our findings highlight the impact of race on treatment response in pediatric anterior uveitis and underscore the need for further research to better understand and address these disparities.

Although ADA is safe and effective in managing pediatric uveitis, evidence suggests decreased efficacy over time. Bravo-Ljubetic et al. reported a mean time to achieve a response of six weeks in 86% of children with uveitis managed with ADA. Sustained remission was achieved in only 60% of patients after 32 months [27]. This is similar to another study reporting quiescence in 60% of children with uveitis after 40 months [28]. In the present study, 74% of children achieved this after a median follow-up of 31 months. However, this fact should be interpreted with caution since we could complete strict follow-up of all cases only during the first 6 months and in 85% at one year. The latter was due to the COVID-19 pandemic, requiring some children to be followed by their local state rheumatologist or ophthalmologist.

Drug immunogenicity is a well-known risk factor associated with the therapeutic failure of anti-TNF inhibitors, potentially leading to disease relapse [14]. This is particularly true for children with JIA-related uveitis, where the incidence of positive AAAs ranges from 16 to 45% [13–15, 29]. Female gender, a younger age of both JIA and uveitis onset, leflunomide use, active uveitis, a higher grade of uveitis at baseline, and lack of treatment with MTX are deemed risk factors for AAAs development [13–15]. During follow-up, positive AAAs were found in 42% of our tested children who experienced uveitis or arthritis flare-up, with no significant associated risk factors. A similar study of children with non-infectious uveitis associated with multiple etiologies reported a 32% proportion of positive AAAs among tested children [10].

We successfully controlled inflammation with infliximab in three children (two with positive AAAs) who experienced uveitis recurrence. A meta-analysis reported a slightly greater response to ADA than infliximab (87% vs. 72%, *p* = 0.08) in children with uveitis [30]. Ardoin et al. [28] using our same criteria to define inflammatory control, reported a somewhat similar 64% (vs. 78%) proportion of children achieving zero ocular inflammation at 1 year follow-up with infliximab. However, the proportion of children with zero inflammation after 3 months of therapy was higher in our cohort. (70% vs. 44%) [28]. This is consistent with a study by Gallagher et al. reporting similar efficacy between both drugs but a quicker response in the ADA group (3.9 vs. 10 weeks) [31].

ADA was safe and effective in managing children with uveitis; however, the study’s retrospective nature, sample size, and heterogeneity of the uveitis etiology represent confounding factors that preclude establishing generalizations. There is always a “referral” bias to tertiary eye care centers, such as Duke Eye Center, leading to the attention of more severe cases, with most of them previously treated by other physicians. Hence, treatment failure and the development of ocular complications could have occurred over time. Strengths include that all children were managed under uniform standards of care by the same specialists (C.E.R and V.L.P).

## Conclusions

Pediatric CAU is a sight-threatening condition that necessitates treatment adjustments based on the severity and activity of the inflammation. Overall, this study found that ADA represents a therapeutic option in patients with refractory pCAU, with the majority achieving remission without significant relapse and/or complication rates. That said, African American patients demonstrated worse baseline disease, significantly higher rates of non-remission, and most of the patients that had recurrence of inflammation. This highlights the importance of additional investigations to understand and address potential disparities in different populations to improve outcomes. No factors were found to be significantly associated with development of AAAs at this sample size. Larger, prospective, and multicenter studies would allow for identification of more valid trends regarding the use of ADA in pCAU.

## Data Availability

No datasets were generated or analysed during the current study.
